# Bodily Deformities

**Published:** 1906-09

**Authors:** 


**Bodily Deformities.** By the late E. J. Chance. Edited by John Poland. Second Edition. Vol. I. Pp. xlviii., 315. London: Smith, Elder & Co. 1905.

This covers a series of lectures delivered by the late Mr. Chance in the year 1852 and subsequently at the City Orthopædic Hospital, including many illustrations drawn by the late author "on the wood" from casts of cases, and brought up to date by the editor. It is written in a pleasant scientific style, and we note quotations from the late Dr. J. C. Prichard, of Bristol, on *Researches into the Physical History of Mankind*, amongst other interesting references. After an introduction bearing upon the origin of specialism, with its early controversy, the anatomy of bodily form and deformity are discussed; also the various causes of deformity. Particularly interesting are the very scientific chapters on the effect of hereditary influence and the causes of deformity acting on the embryo. The remainder of the book is concerned with acquired deformities. We took up the book thinking its perusal would be as dull as most of the orthopædic writings of the present day; we laid it down again as we would a classic, regretting that such literature is so rare, and with thanks to the editor for preserving such a work for the profession.

